# Theoretical approaches for understanding the self‐organized formation of the Golgi apparatus

**DOI:** 10.1111/dgd.12842

**Published:** 2023-02-14

**Authors:** Masashi Tachikawa

**Affiliations:** ^1^ Graduate School of Nanobioscience Yokohama City University Yokohama Japan; ^2^ PRESTO, Japan Science and Technology Agency Chiyoda‐ku Japan

**Keywords:** organelle, physical simulation

## Abstract

Eukaryotic cells fold their membranes into highly organized structures called membrane‐bound organelles. Organelles display characteristic structures and perform specialized functions related to their structures. Focusing on the Golgi apparatus, we provide an overview of recent theoretical studies to explain the mechanism of the architecture of the Golgi apparatus. These studies are classified into two categories: those that use equilibrium models to describe the robust Golgi morphology and those that use non‐equilibrium models to explain the stationarity of the Golgi structures and the constant streaming of membrane traffic. A combinational model of both categories was used for computational reconstruction of the de novo Golgi formation process, which might provide an insight into the integrated understanding of the Golgi structure.

## Graphical abstract

We provide an overview of recent theoretical studies to explain the mechanism of the architecture of the Golgi apparatus. Two classes of theoretical studies are described: equilibrium models to describe the robust Golgi morphology and non‐equilibrium models to explain the stationarity of the Golgi structures and the constant streaming of membrane traffic.

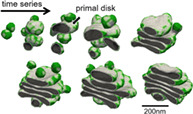

## 1 INTRODUCTION

The endomembranes of eukaryotic cells fold into a variety of highly organized structures called membrane‐bound organelles. The endoplasmic reticulum (ER), Golgi apparatus, mitochondria, and autophagosomes are examples of this. Each organelle displays a characteristic morphology and performs specialized functions related to its morphology (Mullins, 2005). Fundamental questions arise regarding their morphology: How are these shapes formed and maintained? How do their structures and behaviors work as functions of their specific subjects? Do they have the most effective forms for their imposed functions? Accumulating knowledge of molecular biology and biochemistry has largely revealed the molecules necessary for the formation and function of organelles (Klute et al., 2011). Now we can investigate the mechanisms by which these molecules control their structures.

Here, we provide a brief review of theoretical approaches to consider the mechanism of organelle formation. As a membrane‐bound organelle is an assembly of molecules, its behavior is governed by physical rules (Phillips et al., 2013). Thus, physics‐based models are efficient for exploring the mechanism of organelle morphology. We limited the topic of this review to the Golgi apparatus. The Golgi apparatus is a membrane‐bound organelle with a characteristic shape (Klumperman, 2011; Klute et al., 2011). In most species, the minimal functional unit of the Golgi apparatus is called the Golgi stack or mini‐stack. A mini‐stack is generally composed of several flattened membrane sacs of discoid shapes (each of which is called a cisterna) stacked together. The size of the cisterna is approximately 1 μm in diameter and 20 nm in thickness (Klumperman, 2011), and the number of cisternae varies from 3 to 20 depending on the species and states of cells (Day et al., 2013). While several Golgi mini‐stacks are distributed in plant cells and non‐neuronal *Drosophila* cells, in mammalian cells, all Golgi mini‐stacks are gathered in the vicinity of the nucleus and laterally connected; the resulting structure is called a Golgi ribbon (Nakano, 2022). However, some protozoa contain only a single Golgi mini‐stack inside the cell, and the division of the mini‐stack is strictly controlled by cytokinesis (Pelletier et al., 2002). Although the distribution varies among species, the basic structure of the mini‐stack is conserved across almost all species of eukaryotes. Therefore, the basic architecture of the Golgi mini‐stack is assumed to be linked to Golgi function. A famous exception is *Saccharomyces cerevisiae*, whose cisternae are independent of each other and dispersed within the cell (Nakano, 2022).

While the structure of a mini‐stack is quite robust in evolution and individual cells, the Golgi apparatus shows highly dynamic behaviors in the realization of its function (Polishchuk & Mironov, 2004). It is located in the middle of the secretory pathway (membrane trafficking), and various proteins pass through the Golgi apparatus. The main function of the Golgi apparatus is the post‐translational modification and sorting of these proteins. Vesicles budded from the ER, containing immature proteins, are transported to the *cis*‐face of the Golgi apparatus. These proteins are processed and sorted while passing through the Golgi apparatus and shipped to destinations such as the extracellular environment, plasma membrane, and endosome. To carry out this sophisticated process, the Golgi and cisternae are differentiated into three states: *cis*, medial, and *trans*‐Golgi (Glick & Nakano, 2009). Differences in molecular components characterize these states (Tojima et al., 2019). Two hypotheses are proposed for the transport of proteins through the Golgi apparatus: the vesicular transport hypothesis proposes protein transport from early to late cisternae within transporting vesicles, and the cisternal maturation hypothesis proposes the anterograde movement of cisternae, while the retrograde transport of Golgi resident proteins occurs within vesicles (Glick & Nakano, 2009). Real‐time imaging studies of Golgi cisternae in yeast have revealed the clear existence of the cisternal maturation mode in protein transport (Losev et al., 2006; Matsuura‐Tokita et al., 2006). However, a recent study on mammalian cells indicated the predominance of vesicular transport mode (Dunlop et al., 2017). It is supposed to be a mixture of several transport modes, depending on the cell state, to maintain the effective Golgi structures suitable for the states.

To explain the two seemingly incompatible aspects of the structure of the Golgi apparatus, the robust mini‐stack morphology and the stationary streaming of proteins under the membrane traffic process, theoretical studies using two distinct approaches have been developed. The former aspect was mainly investigated using equilibrium models, in which free energies were defined and stable morphologies were described as the minimum energy states. The latter aspect was investigated using non‐equilibrium models with rate equations to directly describe the progress of the detailed membrane traffic process. Studies on de novo construction of mini‐stack morphology have used mixed models containing both equilibrium and non‐equilibrium processes. In this mini‐review, we provide an overview of these approaches and discuss possible directions to unify the understanding of different classes of models.

## 2 EQUILIBRIUM MODELS FOR THE STABILITY OF THE MINI‐STACK MORPHOLOGY

**FIGURE 1:**
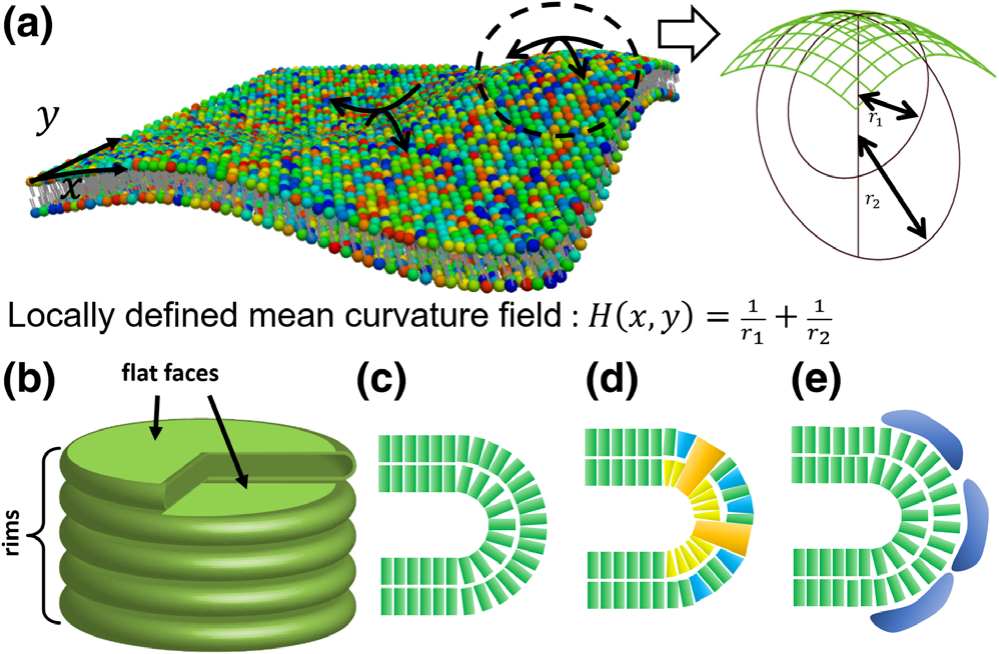
(a) A schematic figure of a field of mean curvature that is the sum of inverse radii of two inscribed circles orthogonal to each other. (b) Simplified‐shape models of discoid‐shaped cisternae and Golgi mini‐stack. (c–e) A close‐up of rim cross‐sections for the area differential elasticity model (c), the nonuniform molecular distribution model (d), and the peripheral protein model (e)

In general, the physical properties of the static state of a focal object are well characterized by the free energy stored by the object. To describe the morphology of the Golgi mini‐stack, the free energy should first be formulated as a function of the morphology. The source of energy that is most closely related to the mini‐stack shape is the bending energy of the lipid membrane. Helfrich (1973) proposed the phenomenological bending energy for lipid membranes as:

$$F_{B}=\frac{\kappa }{2}\int {\left(H-H_{0}\right)}^{2}\mathrm{da},$$

where H is the locally defined mean curvature of the membrane (Figure 1a), and κ and $H_{0}$ are the bending modulus and spontaneous curvature (preferred curvature), respectively. The local form of the free energy is analogous to that of a spring, which is defined by the square of the difference between the actual and equilibrium lengths. The molecular composition of the membrane affects κ and $H_{0}$ values. Thus, they can be locally determined depending on local variations in the molecular compositions.

Let us first consider the single cisterna of a Golgi mini‐stack. For simplicity, we assumed that it is a simple closed membrane without a hole. For such a membrane with a uniform bending modulus and zero spontaneous curvature ($\kappa =\text{const}.,H_{0}=0$), the shape minimizing the bending energy is spherical, as is frequently observed for vesicles. Additional mechanisms are required to stabilize the cisternal discoid shape. The discoid shape is decomposed into three parts: two flat faces and one curved rim (Figure 1b). The rim has a much higher curvature compared with a spherical vesicle with the same membrane area and raises the total bending energy without some rim‐stabilizing mechanisms. Three mechanisms have been proposed to stabilize the rim curvature. The first is the area difference between the inner and outer leaflets of the cisterna lipid membrane, due to the imbalance in the amount of lipid molecules between the leaflets (Jarić et al., 1995). Since the outer leaflet area of the highly curved membrane is larger than that of the inner leaflet area, although both areas of the flat membrane are the same, the area difference affects the curvature. This effect is described by the energy penalty for the deviation of the area difference (ΔA) from the preferred area difference ($\Delta A_{0}$),

$$F_{\mathrm{ad}}=\frac{\kappa_{r}}{2Ad^{2}}{\left(\Delta A-\Delta A_{0}\right)}^{2}\;\text{with}\;\Delta A=2d\int \mathrm{Hda},$$

where A, d, and $\kappa_{r}$ are the total membrane area, membrane thickness, and nonlocal bending modulus, respectively. The model that considers the bending energy and this energy penalty is called the area differential elasticity (ADE) model.

$$F_{\mathrm{ADE}}=F_{B}+F_{\mathrm{ad}}.$$

The preferred area difference is another physical parameter that depends on the membrane molecular composition. Depending on the value, the ADE model has proved a vesicle to show various shapes, including discoid shapes (Jarić et al., 1995). Derganc et al. introduced the ADE model to explain Golgi mini‐stack shapes (Figure 1c, Derganc et al., 2006). Although directly measuring the actual area difference is difficult, assuming that a cisterna is formed from an assembly of vesicles without the exchange of molecules between the inner and outer leaflets, there can be a large difference in the area. For example, if the diameter of a vesicle is 50 nm with a width of 5 nm, the area difference in a typical cisterna is approximately 36% of the total membrane area. Although flip‐flop activity would reduce this large difference, it is not surprising that the difference in the initial state affects the morphology of the cisterna.

Another model to stabilize the rim curvature introduces non‐uniform distributions of molecular components on vesicles, the proportions of which change the spontaneous curvature (Figure 1d, Derganc, 2007, Iglic et al., 2004, Sakai et al., 2020). The conical or inverted conical shape of lipids and membrane‐integrating proteins is known to affect the spontaneous curvature of the membrane. The assembly of these molecules on the cisternal rim can increase spontaneous curvature and stabilize a convex‐shaped membrane. These molecules are known as curvature generators. This situation is described by the spatial variation of $H_{0}$ in the bending energy function ($F_{B}$). In addition, the entropic effect for non‐uniform molecular distribution should be considered (Derganc, 2007, Sakai et al., 2020). Molecular distributions are always forced to approach a uniform state as an entropy‐maximizing effect. For example, considering a curvature‐generating molecule whose area fractions in the flat faces and rims are $\phi_{f}$ and $\phi_{r}$, respectively, the mixing entropy is

$$S_{\mathrm{mix}}=k_{B}\;\sum_{i=r,f}\left\{\phi_{i}\;\log\left(\phi_{i}\right)+\left(1-\phi \right)\log\left(1-\phi \right)\right\}A_{i},\;\text{with}\;\phi_{f}A_{f}+\phi_{r}A_{r}=\text{cont}.$$

where the $A_{f}$ and $A_{r}$ are the total flat face and rim areas, respectively, and $k_{B}$ is the Boltzmann constant. Combined with the bending energy, we obtain the model free energy, including the nonuniform molecular distribution

$$F_{\mathrm{NMD}}=F_{B}\left(H_{0}\left(\phi \right)\right)-S_{\mathrm{mix}}T$$

where T is the absolute temperature and the dependency of the spontaneous curvature on the distribution of the curvature‐generating molecule is explicitly displayed. Iglic et al. introduced the deviatoric term of nonuniform molecular distribution and showed that an axisymmetric vesicle with a specific physical condition displays a discoid shape as the equilibrium shape (Iglic et al., 2004). Delganc explicitly introduced mixing entropy and investigated the relationship between the discoid shape and segregation of curvature‐generating molecules (Derganc, 2007). Phospholipase A2 and lysophosphatidylcholine, a conical lipid produced by phospholipase A2, are candidates for curvature generators (Ha et al., 2012).

Peripheral proteins can also act as membrane curvature generators for the membrane (Figure 1e, Campelo et al., 2017, Tachikawa & Mochizuki, 2017). In this case, the proteins cycle between the cytosol and the membrane instead of undergoing lateral segregation within the membrane. Considering a single peripheral protein acting as a curvature generator with an area fraction of ϕ, the free energy model can be written as

$$F_{\mathrm{PP}}=F_{B}\left(H_{0}\left(\phi \right)\right)-\mu_{\mathrm{cyt}}\phi A_{0}$$

where $\mu_{\mathrm{cyt}}$ is the normalized free energy difference due to protein attachment and $A_{0}$ is the total membrane area. Note that $\mu_{\mathrm{cyt}}$ depends on the chemical potential difference of the protein. Tachikawa and Mochizuki applied this model to a dynamic triangulation membrane (DTM) simulation and demonstrated equilibrium discoid shapes for a wide range of physical parameters (Tachikawa & Mochizuki, 2017). Coatomer proteins can act as peripheral curvature generators of Golgi cisternae (Jackson, 2014). COP I coats were observed at the periphery of the *cis* and medial Golgi cisternae, and a Clathrin coat was observed at the periphery of the *trans*‐Golgi cisternae. Interestingly, the reduction of sphingomyelin in the Golgi membrane removed Clathrin from the *trans*‐Golgi and caused curling of the *trans*‐cisternae. This curling is attributed to the gain of higher curvature energy at the rim of the *trans*‐Golgi cisternae owing to the removal of Clathrin (Campelo et al., 2017).

Stacking is an important feature of Golgi mini‐stack building from an assembly of cisternae (Figure 1b). The flat faces of the cisternae adhere to each other to form a stable mini‐stack. Golgins and GRASPs are families of proteins that act as membrane‐tethering factors to hold the mini‐stack (Ramirez & Lowe, 2009). Adhesion forces can also modify the shape of the Golgi mini‐stack. Derganc et al. proposed a variable cisternal size model, where the total amount of the mini‐stack membrane area is conserved, and found that a change in adhesion strength can modify the size and number of cisternae (Derganc et al., 2006). They estimated 8,000 tethering molecules per square micrometer with binding energy of $5\;k_{B}T$ for a typical Golgi structure with seven cisternae. This result may shed light on a fundamental problem of Golgi morphology in controlling the number of cisternae.

## 3 NON‐EQUILIBRIUM MODELS FOR THE CONSTRUCTION OF THE MINI‐STACK MORPHOLOGY

Free energy‐based physical modeling indicates that two effects, the curvature stabilizing factor and the cisternal stacking force, are necessary for the formation of a stable Golgi mini‐stack. In these previous studies, the discoid and mini‐stack shapes were provided as the initial conditions to examine their stabilities. The next question should be addressed in the construction of Golgi mini‐stacks. The formation of the Golgi apparatus is frequently observed in cells. In plant cells, the Golgi mini‐stacks are newly formed along with the growth of the cell (Hawes et al., 2010). During cell division in mammalian cells, the Golgi apparatus is decomposed into small vesicular fragments, and after cell division, these vesicles assemble and reform their characteristic Golgi shape (Lucocq & Warren, 1987). In contrast to the case of plant cells, all vesicles were provided from the beginning of the process and spread throughout the cytosol. The Golgi reassembly process completes in about 10 min (Rabouille et al., 1995). What does the remarkable difference in the formation processes mean for the outcome when a common basic architecture is present? One possibility is that the basic architecture of the Golgi mini‐stack is so stable that species have flexibly altered the formation process over the course of evolution without changing the morphology of the product. In other words, under the mechanism of stabilizing the Golgi mini‐stack shape, with a type of free energy proposed above, the Golgi mini‐stack shape may be formed spontaneously without minute control.

**FIGURE 2:**
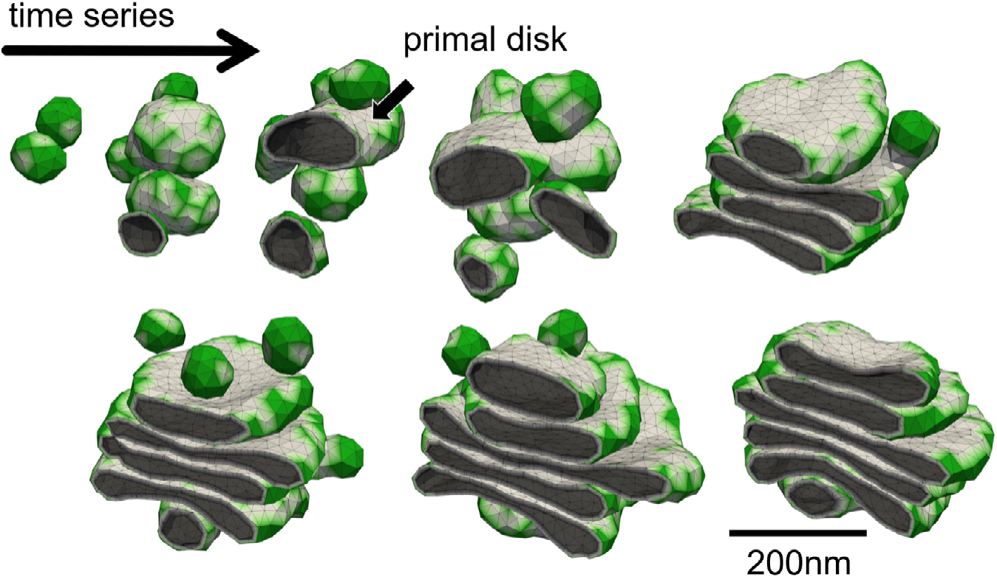
Snapshots of the Golgi reassembly simulation shown in the temporal order. Primal disk is indicated

Tachikawa and Mochizuki (2017) examined the spontaneous construction of the Golgi mini‐stack shape from an assembly of vesicles using computer simulation. They assumed a situation resembling the Golgi reassembly process in mammalian cell division. In vitro reconstruction of the process (Tan et al., 2010) strongly indicates that the process proceeds purely under physicochemical rules. The free energy, consisting of the bending energy with peripheral curvature generator molecules and intermembrane adhesion energy, was adopted for the model stabilizing the Golgi mini‐stack shape. Additionally, the fusion process between two membranes facing each other was introduced. The fusion probability was set to depend on the membrane curvature, and had higher values for the convex‐shaped membrane. This is a model assumption based on the observation of the fusion process in the interphase Golgi; fusion frequently occurs at the curved rim region and is supposed to be rare in the interior region of the Golgi mini‐stack. Because membrane fusion is catalyzed by soluble *N*‐ethylmaleimide‐sensitive‐factor attachment protein receptor (SNARE) proteins, which release the stored free energy in the process, the model fusion process was set to proceed unidirectionally as a non‐equilibrium process (fission process was not incorporated in the simulation). DTM simulations, which describe the membrane shape with a triangular polygon, were performed to ensure high variability of the membrane shapes. The Monte‐Carlo method was used in this study. Several snapshots of the time course of the Golgi reassembly simulation are shown in Figure 2. During vesicle assembly, a fused vesicle reaching a certain size turned into a discoid shape. The primarily formed disk acted as a “crystal nucleus,” and a layered structure resembling a Golgi mini‐stack was constructed around the primal disk. This is a self‐organization process in which an assembly of vesicles obeying physical rules spontaneously forms a highly organized structure. Due to the limitation of the computational resource, the outcome structures were smaller compared to actual Golgi mini‐stacks. However, the stable construction process observed in the simulations indicate that the longer simulations may generate structures comparable to actual Golgi mini‐stacks.

The study has revealed some physical conditions for the formation of a clear mini‐stack‐like shape (Tachikawa & Mochizuki, 2017). Typical mechanical parameter values which generate fine‐layered structures are presented in Table 1. Fusion restriction within an appropriate membrane curvature range (comparable to the cisternal rim curvature) is necessary for this formation. The weaker restriction generated highly connected sponge‐like shapes, the stronger restriction suppressed the occurrence of fusion events, and the resulting structures were fragmented. The membrane adhesion force should be weak; stronger adhesion enhances entanglement among cisternae and generates nested structures. It is one‐twentieth smaller than the estimation in Derganc et al., 2006. In the simulations, vesicles were added one by one in a prepared space in an interval, which indicates the density of the vesicles. A shorter interval, which indicates a higher vesicle density, generated partially broken mini‐stack shapes. This indicated that sufficient relaxation of shapes is necessary in each step of the growth of membrane compartments by vesicle fusion for the formation of a clear mini‐stack shape, and the fast accumulation of vesicles blocked relaxation. Although it is difficult, in principle, to determine the exact timescale of the Monte‐Carlo simulations, the actual shape‐relaxation timescale (milliseconds) and the timescale of the completion of Golgi reassembly (~10 min; Rabouille et al., 1995) seem to be sufficiently separated.

**TABLE 1:** Typical mechanical parameter values to generate fine‐layered structures used in Tachikawa and Mochizuki (2017)

Physical arameters	Value
Bending modulus	$20\;\left[k_{B}T\right]$
Osmotic pressure difference	$6.0\sim 7.0\times 10^{-4}\;\left[\mathrm{atm}\right]$
Protein adsorption energy	$7.0\times 10^{-3}\;\left[k_{B}T/{\mathrm{nm}}^{2}\right]$
Intermembrane adhesion energy	$2.0\times 10^{-3}\;\left[k_{B}T/{\mathrm{nm}}^{2}\right]$
Spontaneous curvature	$4.0\times 10^{-2}\;\left[1/\mathrm{nm}\right]$
Curvature threshold for fusion restriction	$5.0\times 10^{-2}\;\left[1/\mathrm{nm}\right]$

Kuhnle et al. (2010) introduced another interesting coarse‐grained model to investigate the biogenesis of the Golgi mini‐stack, in which a vesicle is represented by a sphere and larger membrane compartments are described by numerous connected vesicular spheres. For example, a discoid shape is formed by a layer of laterally connected spheres. Although this model cannot calculate membrane bending energy, a low‐cost simulation can generate large membrane structures. The study demonstrated the de novo biogenesis of the Golgi mini‐stack from vesicles sent from one direction, mimicking vesicular transport from the ER exit site.

## 4 NON‐EQUILIBRIUM MODELS FOR THE STEADY STATE OF MEMBRANE TRAFFIC THROUGH GOLGI

The Golgi apparatus is a pathway for proteins to be secreted or integrated into the plasma membrane, and some modifications are applied to the proteins in a regulated order while passing through it. From this viewpoint, it is natural to ask how stationary structures are formed in the middle of streams of substances. The structure of the Golgi apparatus, as well as the regulated order of enzymes for protein modification in the cisternae, are to be questioned. Several studies have attempted to elucidate the mechanism of stream‐induced stationary structures.

Rate equations analogous to chemical reactions are mainly used as model equations to describe the change in the amount of proteins and membrane compartments (Gong et al., 2008; Ispolatov & Musch, 2013; Sens & Rao, 2013). Because the speeds of state transitions are described by parameters in the rate equation, these models are completely non‐equilibrium models. This is in contrast to the previously mentioned non‐equilibrium model for the construction of the mini‐stack morphology, in which the construction was described by a relaxation process in the landscape of the equilibrium free energy, although the vesicle addition process and membrane fusion rule were modeled in a non‐equilibrium manner (Tachikawa & Mochizuki, 2017). The basic architectures shared among these models are as follows: (1) unit vesicles are introduced into the prepared space at a constant rate; (2) vesicles fuse with each other to generate larger compartments or fuse to the pre‐existing compartments; and (3) the fission process generates vesicles from the compartments. (4) Vesicles were removed from the space. The reactions between vesicles and membrane compartments are described as rate equations, and hence, the amounts of membrane compartments are indicated by variables in these models.

In some models, vesicles have states (*cis*, medial, or *trans*) identified by the presence of molecules (Ispolatov & Musch, 2013; Sachdeva et al., 2016; Vagne et al., 2020). In these models, the proportions of identified molecules in a single compartment are the model variables. These states can be converted from *cis* and medial to *trans*, represented by the rate equation. The fusion, fission, and removal rates depend on the states. In many cases, the prepared spaces are dimensionless, and two compartments can react regardless of the distance between them (a well‐mixed condition or a mean field model in physics terminology), although some models introduce a one‐dimensional space that spans from the ER to the plasma membrane (Dmitrieff et al., 2013; Sachdeva et al., 2011; Sachdeva et al., 2016).

The most important concern of these models is the range of physical conditions in which membrane compartments of finite sizes exist (Gong et al., 2008; Sachdeva et al., 2011). These studies have revealed that inadequate physical conditions can unboundedly accumulate membrane compartments or shrink them. Dmitrieff et al. (2013) compared the model behaviors and fluorescence recovery after photobleaching experiments (FLAP) for cargo proteins and concluded that neither of the modes can explain the stream of cargo proteins. These results indicate the necessity of controlling the membrane trafficking rates for stable stationary states. The transition of membrane states has also been described by various models; the autonomous progression of states was supposed to represent the Rab cascade reaction (Sachdeva et al., 2016, Vagne et al., 2020), and the gradual crossing of SNARE activities was also considered (Ispolatov & Musch, 2013).

## 5 FUTURE OUTLOOK

Here, we provide an overview of recent theoretical studies on the architecture of the Golgi apparatus. Reflecting the bilateral character of the Golgi apparatus, two classes of theoretical models have been proposed: equilibrium models to describe the robust morphology of the Golgi mini‐stack and non‐equilibrium models to explain the stationarity of Golgi structures during the constant streaming of membrane traffic. The building blocks of the models are also different. The former studies combined several free energies to represent the focal situation, and the latter used rate equations for fusion and fission reactions among membrane compartments. To gain further insight into the architecture of the Golgi apparatus, a combination of these models is necessary. Non‐equilibrium models for describing the formation process of the mini‐stack morphology may pioneer studies on this combination (Kuhnle et al., 2010; Tachikawa & Mochizuki, 2017). The reaction processes of membrane traffic can be incorporated into them. However, these models were further simplified compared with the actual systems. For example, directional transport of vesicles due to motor proteins is not considered, but is an important physical process to describe Golgi morphology in the process of the stationary secretion pathway. The effect of crowded conditions of the cytosol on the morphology and behavior of membranes was not considered. A number of chemical steps within the fusion, fission, and cisternal maturation processes were also ignored. Undoubtedly, the actual situation of cells is so complex that not all details of the cellular processes can be incorporated. A suitable simplification of the model based on an adequate biological question is necessary to elucidate the mechanism of the organelle architecture.
